# Mechanical behavior of tooth-class II restoration complex with various restorative materials using linear and non-linear finite element analysis

**DOI:** 10.1038/s41598-026-40204-3

**Published:** 2026-02-21

**Authors:** Young-Ho Yu, Mi-Jeong Jeon, Su-Jung Shin, Jeong-Won Park

**Affiliations:** 1 Department of Dentistry, Graduate School of Yonsei University, Seoul, Korea; 2 Department of Conservative Dentistry, College of Dentistry, Yonsei University, Seoul, Korea; 3 Department of Conservative Dentistry, Gangnam Severance Hospital, College of Dentistry, Yonsei University, Seoul, Korea

**Keywords:** Tooth-class II restoration complex, Finite element analysis, Composite resin, Ceramic, Amalgam, Gold, Health care, Materials science, Medical research

## Abstract

This study aimed to investigate the mechanical behavior of the tooth–class II restoration complex restored with composite resin, ceramic, amalgam, and gold using three-dimensional linear and non-linear finite element analyses (FEA). Previous FEA studies generally assumed linear mechanical properties and bonded interfaces between tooth and restorative materials, which differ from clinical reality, particularly for non-bonded restorations such as amalgam and gold. Therefore, in this study, non-linear mechanical properties and non-bonded contact conditions were adopted for different restorative materials. The results showed that the highest enamel and dentin stresses occurred in amalgam restorations, while the lowest were found in ceramic restorations. Amalgam and gold produced higher stress in enamel and dentin compared with composite resin and ceramic. The higher stress observed in amalgam and gold was mainly associated with the non-bonded (frictional) contact condition, because the stresses in these restorations remained below their reported yield strengths under the applied loading conditions. This study provides mechanical insights into the behavior of the tooth–class II restoration complex and offers a possible mechanical interpretation of previously reported clinical observations. These findings should be interpreted within the limitations of the finite element model and do not directly represent clinical outcomes.

**Supplementary Information:**

The online version contains supplementary material available at 10.1038/s41598-026-40204-3.

## Introduction

Recently, various materials have been utilized for tooth restorations in dental clinics, owing to advancements in dental restorative materials and techniques, including bonded materials such as composite resins and dental ceramics, as well as traditional non-bonded materials such as amalgam and gold^1–6^. The selection of restorative materials for dental treatments is one of the most critical factors for preserving the remaining tooth structure and the long-term prognosis of the tooth–restoration complex. Dentists choose restorative materials based on the remaining tooth structure, masticatory force, and oral hygiene maintenance ability. Fracture of the remaining tooth structure and restoration is a major factor affecting the long-term prognosis of the tooth–restoration complex^7–9^.

Various restorative materials can clinically affect the fracture and longevity of the tooth–restoration complex, owing to differences in their mechanical properties and tooth-bonding characteristics^10^. If the restorative material has a lower modulus than enamel or dentin, the structural stiffness of the tooth–restoration complex may decrease, resulting in greater tooth deformation and stress^11^. In addition, the ductility and brittleness of the material are important factors. In the case of ductile materials such as gold, the yielding phenomenon can occur at forces above the yield stress, which can result in greater deformation and stress on the tooth structure. In addition, the bonding characteristics of the restorative material with the tooth significantly affect the deformation and stress transfer characteristics between the restorative material and the tooth^12^. Therefore, non-bonded restorations may decrease the structural stiffness of the tooth–restoration complex compared with bonded restorations, and when flexion occurs in the tooth by lateral forces, greater deformation and stress may be applied to the tooth. Teeth restored with amalgam and gold inlays have been reported to exhibit a higher prevalence of cracking and are more susceptible to cracking than those restored with resin or porcelain inlays^13^. However, the exact mechanism by which non-bonded restorations, such as amalgam and gold inlays, induce tooth cracking has not yet been clearly established.

Therefore, a profound understanding of the mechanical behavior of the tooth-restoration complex is essential to ensure long-term favorable outcomes of restorations and preserve the remaining tooth structure^14–16^. However, the long-term prognosis of tooth–restoration complexes with different restorative materials varies greatly depending on many variables, such as the patient’s individual occlusal force, dietary habits, bruxism, anatomic structure of the tooth, cavity size, and cavity design, making it difficult to control these variables clinically. In addition, identifying clinical differences in restoration materials is time consuming, making it challenging to study these differences in clinical tests or in vitro experiments. Therefore, previous studies have widely adopted finite element analysis (FEA) to study the mechanical behavior of the tooth-restoration complex according to the restorative material.

FEA has been used in numerous studies to investigate the mechanical behavior of tooth–restoration complexes based on restorative materials^17–20^. However, most of these studies have assumed linear mechanical properties of dental materials and perfectly bonded contact conditions between teeth and restorative materials, even when analyzing restorations that clinically behave as non-bonded systems^17–20^. These assumptions are unrealistic. The problem with perfect bonding between dentin and post material in FEA has been pointed out by many clinicians and researchers^21^. Finite element research has also reported that the contact conditions between teeth and restorative materials can increase the fracturing potential of teeth more exponentially than bonded contact conditions^22^. Amalgam and gold exhibited non-linear mechanical properties under compressive stress. In addition, nonbonded restorations have little bonding force, especially perpendicular to the tooth^23–27^. Therefore, in this study, conventional linear FEA was performed for composite and ceramic restorations, which have brittle mechanical properties and high adhesive strength to teeth. Non-linear FEA was performed for gold and amalgam restorations, which have large non-linear mechanical properties and low adhesive strength to teeth.

## Methods

**Fig. 1:**
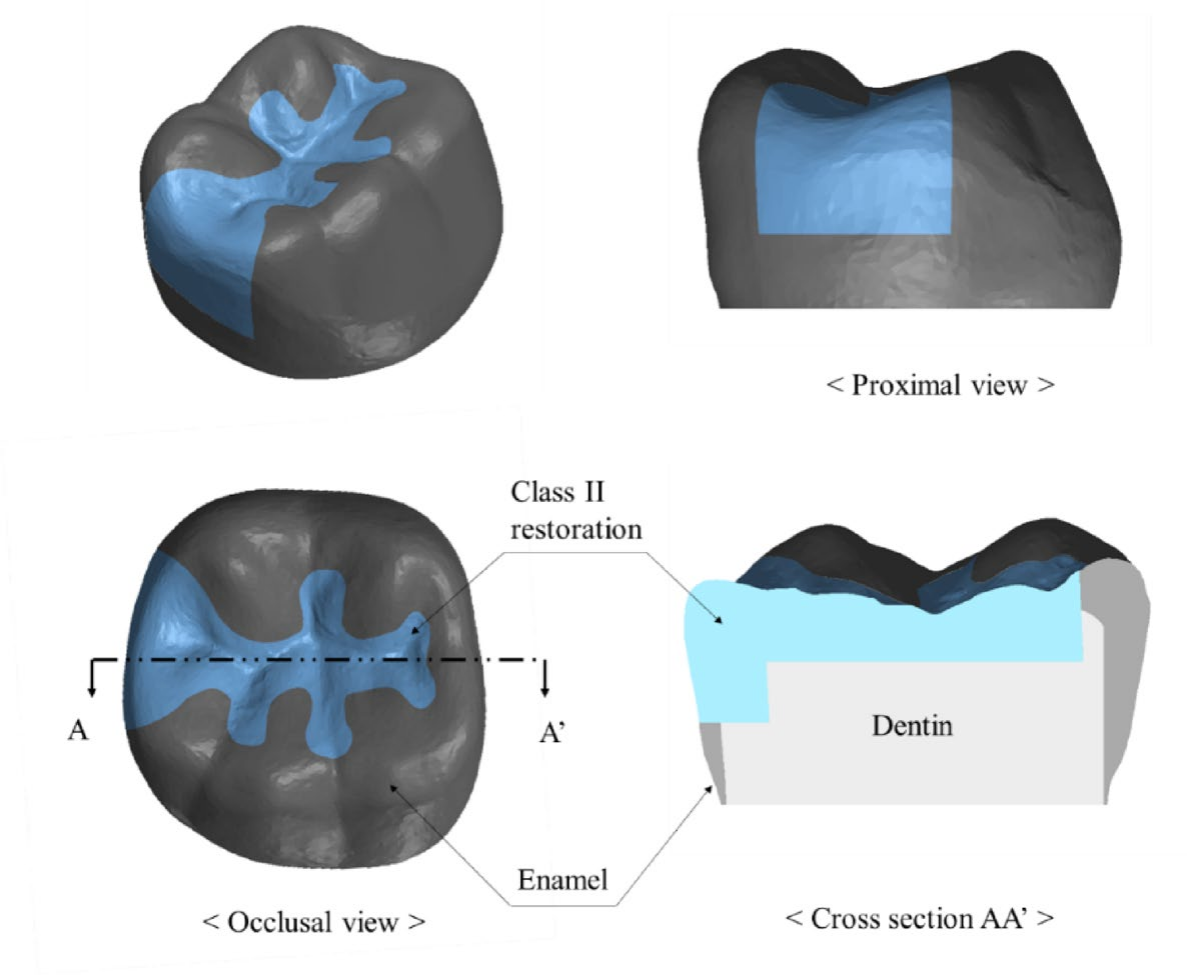
Tooth-class II restoration complex model used in finite element analysis.

**Fig. 2:**
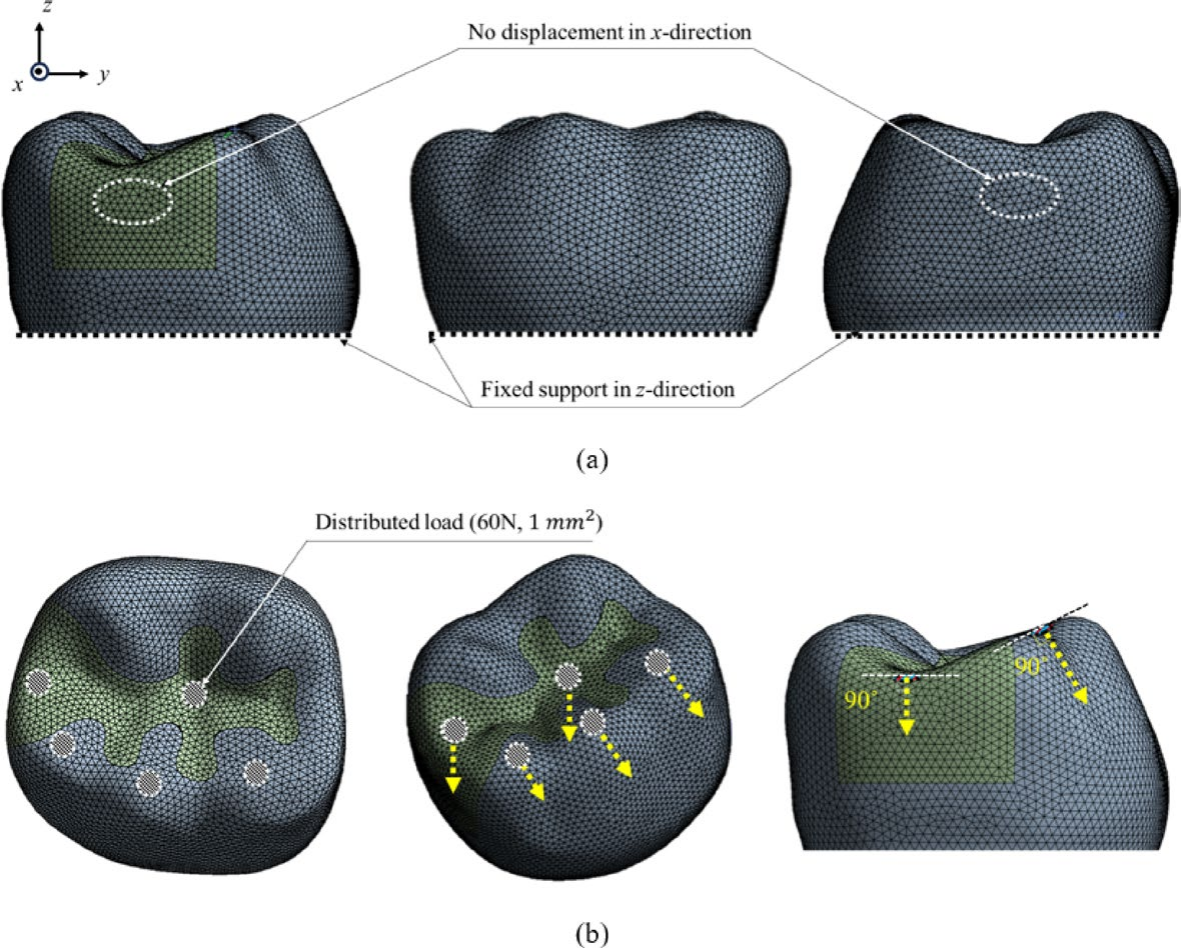
Finite element model of tooth-class II restoration complex. **a** Boundary conditions. **b** Loading conditions.

The mandibular first molar, which is known to withstand the greatest force and fractures in the oral cavity, was used in this study^28^. Because the purpose of this study was to understand the mechanical behavior of the crown portion with various class II restoration materials, only the crown portion was modeled for efficient analysis, as shown in Fig. 1. Intact mandibular first molars were extracted and scanned using micro-CT (Skyscan1076, Skyscan, Konitch, Belgium) to obtain three-dimensional (3D) data. A class II restoration with a 90° cavity margin angle was designed as shown in Fig. 1 using Ansys SpaceClaim^®^ (ANSYS SpaceClaim^®^ version 2021, ANSYS Inc., Canonsburg, PA, USA). After performing surface tessellation and generating a 3D volume using Ansys SpaceClaim^®^, the obtained solid model was transferred to Ansys Mechanical R21^®^ (ANSYS version 2021, ANSYS Inc., Canonsburg, PA, USA) to generate a tetrahedron type of mesh as shown in Fig. 2. The mesh has a quadratic element order, and the average mesh size was set to 280 μm, with 73,264 elements and 125,493 nodes. An appropriate mesh size was determined through a mesh convergence test, and an element size of 280 μm was selected based on the convergence of the maximum principal stress.

**Fig. 3:**
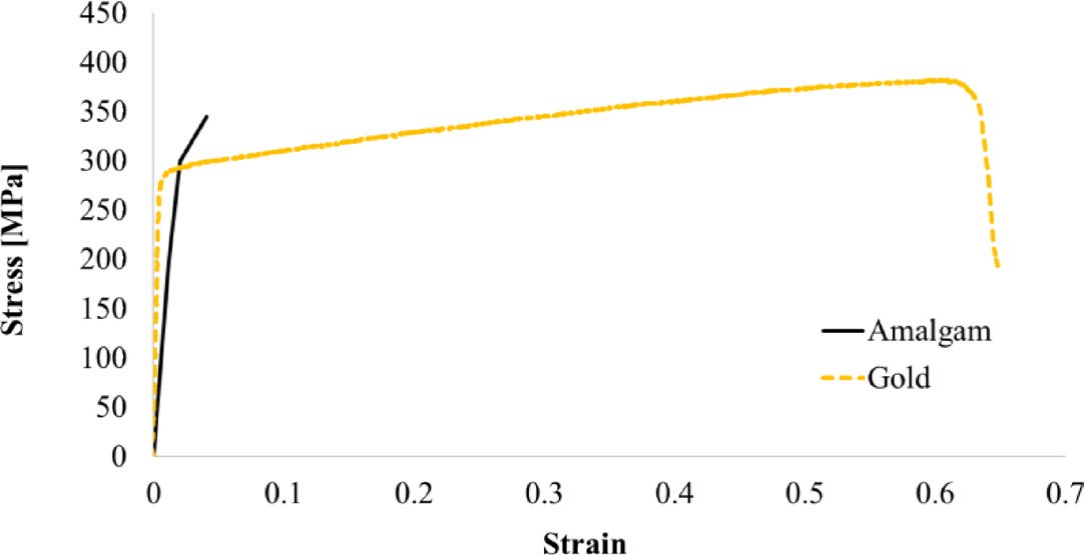
Stress-strain curves of amalgam and gold used for the non-linear material model.

In this study, composite resin, lithium disilicate glass-ceramic, amalgam, and gold restorations were simulated. The mechanical properties assigned to each material were adopted from previously published experimental and numerical studies, as summarized in Table 1. The material properties of the composite resin and lithium disilicate ceramic used in this study were those of Filtek Z350 (Filtek Z350, 3 M/ESPE, St. Paul, MN, USA) and IPS e.max CAD (Ivoclar Vivadent, Schaan, Liechtenstein). Linear mechanical properties and bonded contact conditions with teeth have been adopted for composite resins and ceramics known as bonded restorations. Whereas for amalgam and gold, which is known as non-bonded restoration, non-linear mechanical properties and frictional contact conditions with the tooth are adopted. Additionally, the linear properties and bonded contact conditions were analyzed to compare the effects of these different conditions. The stress-strain curves of the amalgam and gold used in the non-linear FEA are shown in Fig. 3. For amalgam and gold, frictional non-linear contact conditions between the tooth and restorative material were adopted. The contact formulation was set to the Augmented Lagrange formulation in ANSYS Mechanical. The contact condition allows separation and tangential movement of the tooth when tooth-class II restoration is deformed under occlusal force. A friction coefficient of 0.5 was assumed as a representative value for metal–resin contact to reflect the potential presence of an intermediate cement/adhesive layer^29^. Normal contact behavior was defined as hard contact using the augmented Lagrange formulation, preventing overclosure while allowing separation. Initial contact was established using the “Adjust to Touch” option.

**Table 1:** Material properties used in finite element analysis.

Enamel	Young’s modulus [GPa]	Poisson’s ratio	Yield strength [MPa]	Tensile strength [MPa]	Reference
	80	0.33	–	35	^23,24^
Dentin	15	0.31	–	65	^23,24^
Dental gold	85	0.25	280	–	^10,24^
Amalgam	50	0.29	275	50	^24,28^
Composite resin (Filtek Z350)	10	0.27	–	85	^11,28^
Indirect ceramic (IPS E.max CAD)	94.1	0.25	–	–	^1,18^

The total deformation and maximum principal stress applied to the tooth and restoration were calculated for different restoration materials. The boundary and loading conditions used in this study are illustrated in Fig. 2. To represent interproximal support without explicitly modeling the adjacent tooth, displacement at the proximal contact area was constrained only in the mesio–distal (X) direction^30–32^. To prevent rigid-body motion, the basal surface of the crown model was constrained in all directions, as shown in Fig. 2 (a). Distributed loads of 60 N were applied to each of five occlusal contact areas, each with a contact area of 1 mm², resulting in a total occlusal load of 300 N on the mandibular first molar, as shown in Fig. 2 (b) ^33^.

## Results

**Fig. 4:**
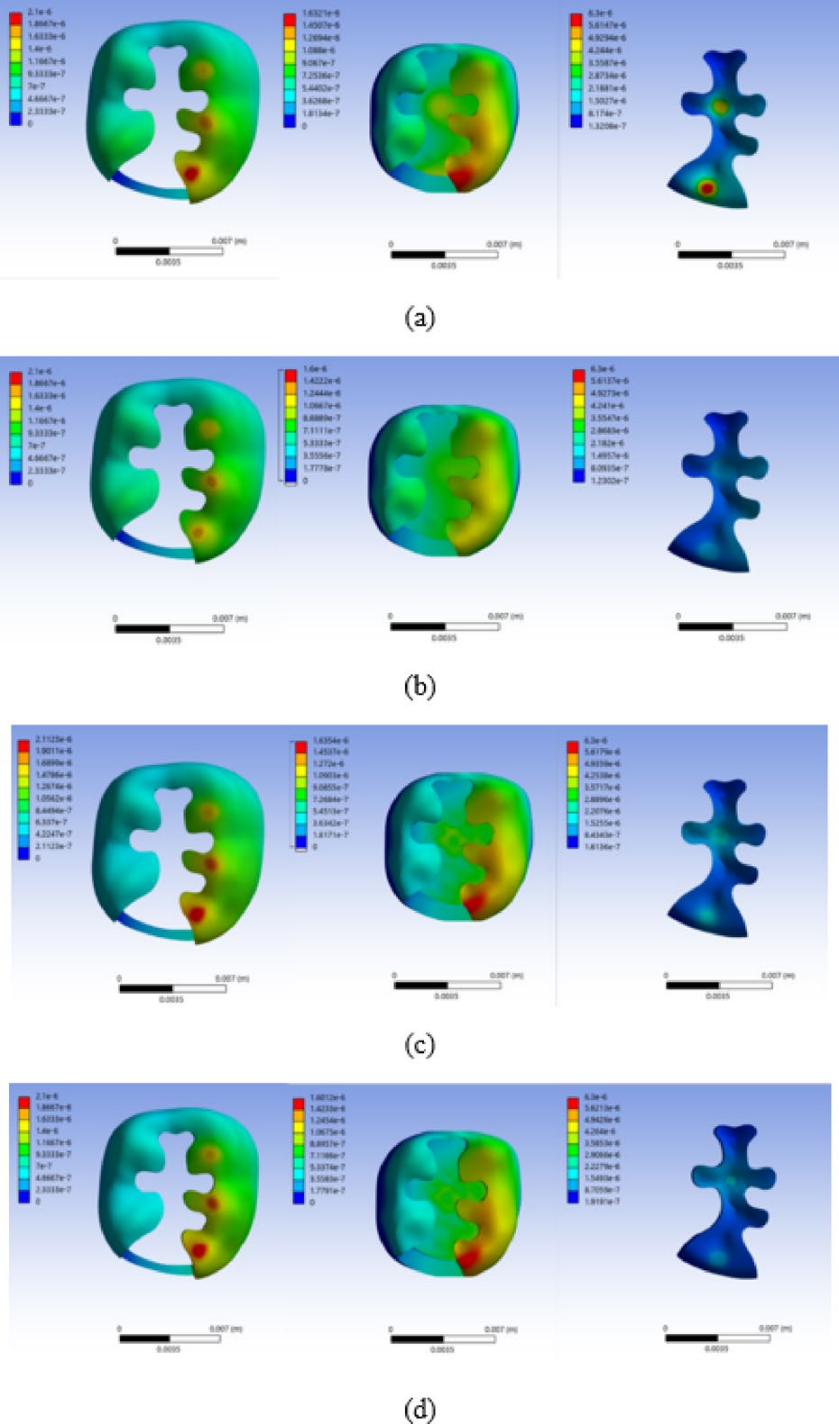
Total deformation distributions in enamel, dentin, and restoration for each restorative material. **a** Composite resin, **b** Ceramic, **c** Amalgam, and **d** Gold. Total deformation is expressed in micrometers (µm). The color scale was adjusted independently for each subfigure to improve visualization; therefore, the numerical deformation ranges differ among (a)–(d). The minimum and maximum values are indicated on the color bar in each subfigure.

**Fig. 5:**
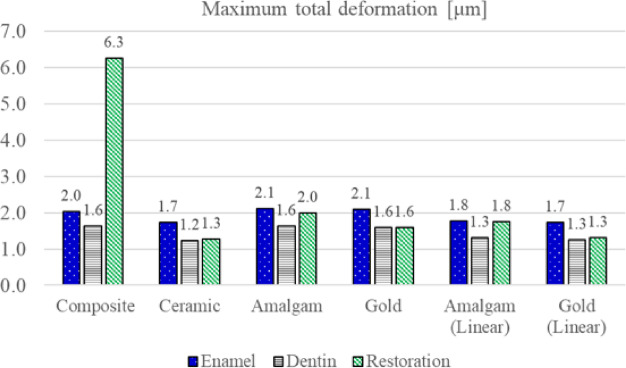
Maximum total deformation of enamel, dentin, and the restoration for each restorative material. The bar graph reports maximum values in micrometers (µm).

Total deformations of enamel, dentin, and restoration, with respect to different restoration materials are shown in Fig. 4. For composite resin, the largest maximum total deformation of the restoration occurred at the proximal area where the occlusal force was applied. For ceramic restoration, the maximum total deformation of the restoration occurred in the central and proximal areas where the occlusal force was applied. For amalgam and gold restorations, the maximum total deformation of the restoration occurred in the central area where the occlusal force was applied. For all materials, the maximum total deformation of enamel and dentin occurred at the proximal box area below the region where the occlusal force was applied. The maximum total deformation of enamel, dentin, and restoration with respect to the restorative materials are shown in Fig. 5. The maximum total deformation in enamel was 2.0, 1.7, 2.1, 2.1, 1.8, and 1.7 μm for composite resin, ceramic, amalgam, gold, amalgam (linear), and gold (linear) restorations, respectively. The maximum total deformation in dentin was 1.6, 1.2, 1.6, 1.6, 1.3, and 1.3 μm for composite resin, ceramic, amalgam, gold, amalgam (linear), and gold (linear) restorations, respectively. The maximum total deformation in the restoration was 6.3, 1.3, 2.0, 1.6, 1.8 and 1.3 μm for composite resin, ceramic, amalgam, gold, amalgam (linear), and gold (linear) restorations, respectively.

**Fig. 6:**
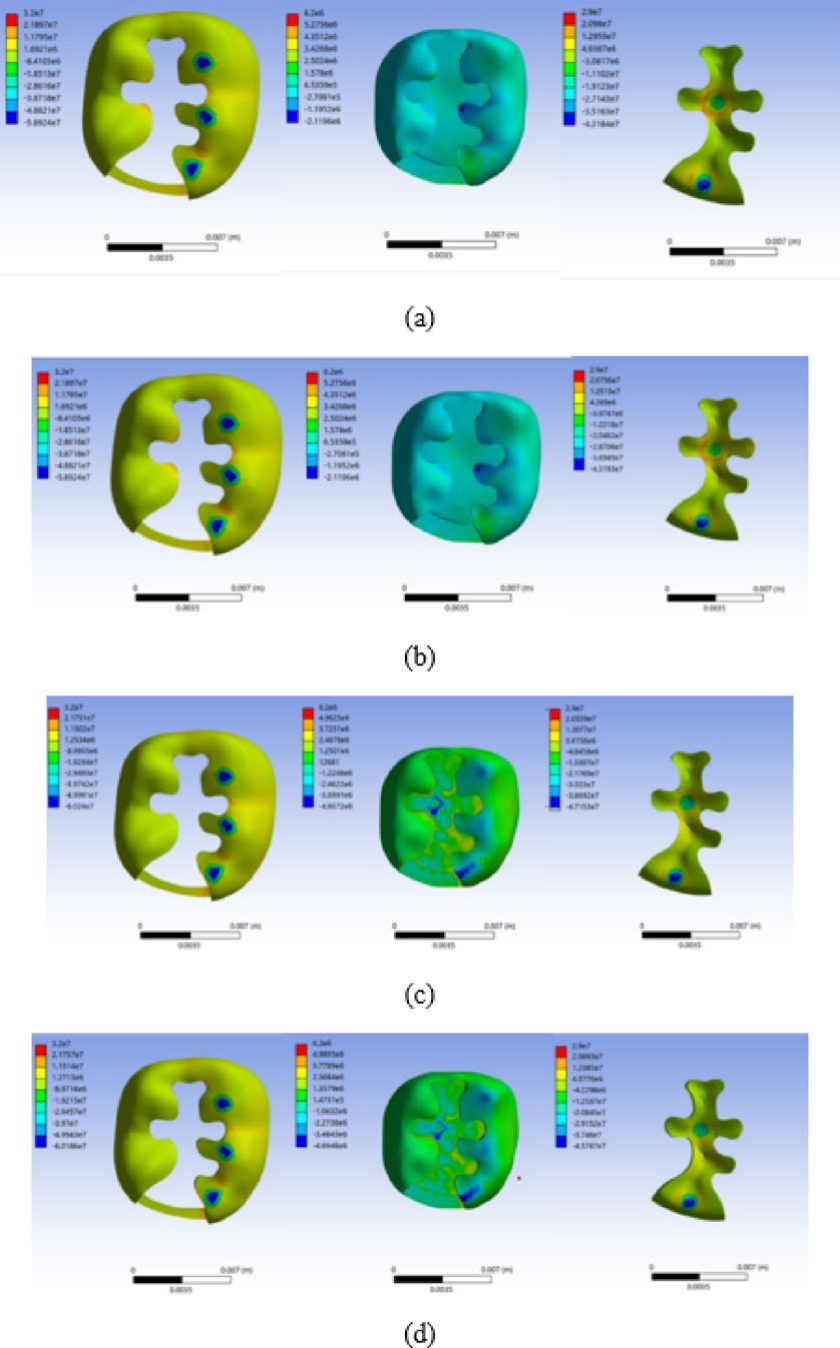
Maximum principal stress distributions in enamel, dentin, and the restoration for each restorative material. **a** composite resin, **b** ceramic, **c** amalgam, and **d** gold. Maximum principal stress is expressed in megapascals (MPa). The color scale was adjusted independently for each subfigure to improve visualization; therefore, the numerical ranges differ among **a**–**d**. The minimum and maximum values are indicated on the color bar in each subfigure.

The maximum principal stress of enamel, dentin, and restoration with respect to the restorative material is shown in Fig. 6. For composite and ceramic, the maximum principal stress of restoration occurred in the area around the region where the occlusal force was applied. For amalgam and gold, the maximum principal stress of restoration occurred at the boundary between the restoration and the tooth beneath the central area where the occlusal force was applied.

For composite, the maximum principal stress of enamel and dentin occurred at the margin of the cavity, close to the central fossa. For ceramic, amalgam, and gold, the maximum principal stress of enamel and dentin occurred at the boundary between enamel and dentin, beneath the area where the occlusal force was applied.

**Fig. 7:**
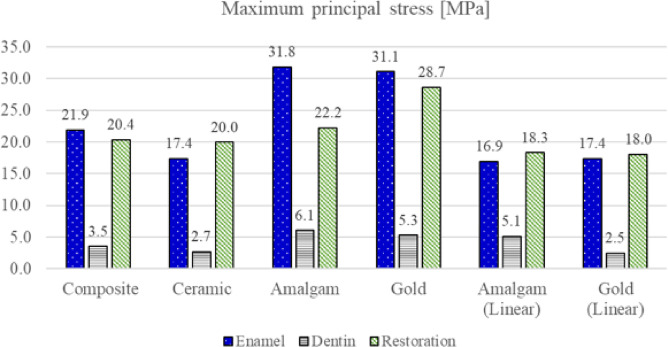
Maximum principal stress of enamel, dentin, and the restoration for each restorative material. The bar graph reports maximum values in megapascals (MPa).

The maximum principal stress of enamel, dentin, and restoration with respect to the restorative material is shown in Fig. 7. The maximum principal stress in enamel was 21.9, 17.4, 31.8, 31.1, 16.9, and 17.4 MPa for composite resin, ceramic, amalgam, gold, amalgam (linear), and gold (linear) restorations, respectively. The maximum principal stress in dentin was 3.5, 2.7, 6.1, 5.3, 5.1, and 2.5 MPa for composite resin, ceramic, amalgam, gold, amalgam (linear), and gold (linear) restorations, respectively. The maximum principal stress in the restoration was 20.4, 20.0, 22.2, 28.7, 18.3, and 18.0 MPa for composite resin, ceramic, amalgam, gold, amalgam (linear), and gold (linear) restorations, respectively.

## Discussion

Restorative materials can affect the fracture and longevity of tooth structures clinically owing to differences in the mechanical properties and bonding characteristics of restorative materials. Amalgam and gold can exhibit non-linear mechanical behavior under high compressive stresses, although the stresses predicted in the present simulations remained below the reported yield strengths under the applied loading conditions. In addition, the bonding characteristics of non-bonded restorations may decrease the structural stiffness of the tooth–restoration complex. In reality, even when good bonding between the tooth and restorative material is obtained, microcracks or debonding can develop at sites where the interfacial stress exceeds the bond strength, and early debonding at the tooth–restoration interface may cause a dramatic change in the stress distribution that can lead to an immediate propagation of the interfacial failure^21^. This early debonding is more likely to occur in non-bonded restorations such as amalgam and gold. Therefore, in this study, we conducted linear and non-linear FEA considering both the non-linear mechanical properties and the non-bonded contact conditions. The total deformation and maximum principal stress applied to the tooth and restoration were calculated for different restoration materials.

The maximum total deformation of restoration was found to be the largest in composite resin, followed by amalgam, gold, and ceramic restorations in descending order. For enamel and dentin, the maximum total deformation was found to be the largest in amalgam and gold, followed by composite resin and ceramic restorations. The largest deformation of composite resin appears to be due to its lowest elastic modulus. In ceramic restorations, the smallest total deformation was observed for enamel and dentin restorations. This seems to be due to the high structural stiffness of the tooth–restoration complex caused by the bonded contact condition, as well as the largest elastic modulus of ceramic restorations.

The maximum principal stress of restoration was found to be the largest in gold, followed by amalgam, composite resin, and ceramic restorations, in descending order. In enamel and dentin, the maximum principal stress was found to be highest in amalgam, followed by gold, composite resin, and ceramic restorations. The highest stress occurred in amalgam restorations, whereas the lowest stress occurred in ceramic restorations. In amalgam restorations, the stress generated in the enamel and dentin was 83% and 127% higher, respectively, than that in ceramic restorations. The stress exerted on enamel and dentin was higher in amalgam and gold restorations than in the composite resin and ceramic restorations.

The higher stresses observed in amalgam and gold were primarily associated with the frictional (non-bonded) contact condition, which permits interfacial separation and sliding. Although non-linear material models were included, the predicted stresses in amalgam and gold remained below the yield strengths, suggesting that contact non-linearity dominated the stress redistribution under the present loading conditions.

In enamel, the stress increased by 88% for amalgam and 79% for gold, whereas in dentin, the stress increased by 19% and 112%, respectively. In addition, these findings provide a possible mechanical explanation for previous clinical reports indicating a higher incidence of cracks in teeth restored with amalgam and gold compared with resin or porcelain inlays; however, direct clinical extrapolation should be made with caution given the limitations of the finite element model^13^. The present analysis focuses on relative mechanical behavior under controlled loading conditions rather than predicting absolute clinical outcomes.

**Fig. 8:**
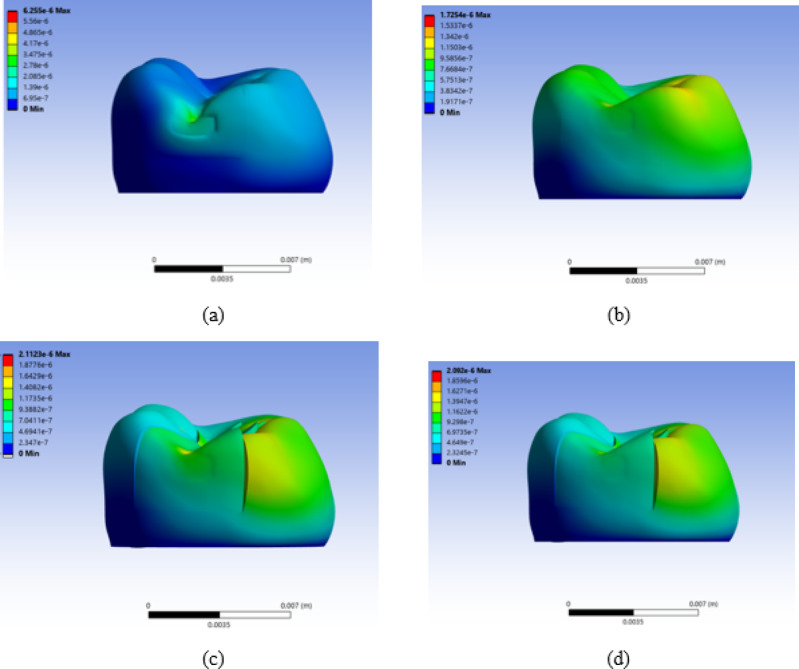
Proximal view of the deformed tooth–Class II restoration complex for each restorative material. **a** Composite resin, **b** Ceramic, **c** Amalgam, and **d** Gold. Total deformation is expressed in micrometers (µm). The color scale was adjusted independently for each subfigure to improve visualization; therefore, the numerical ranges differ among (a)–(d).

**Fig. 9:**
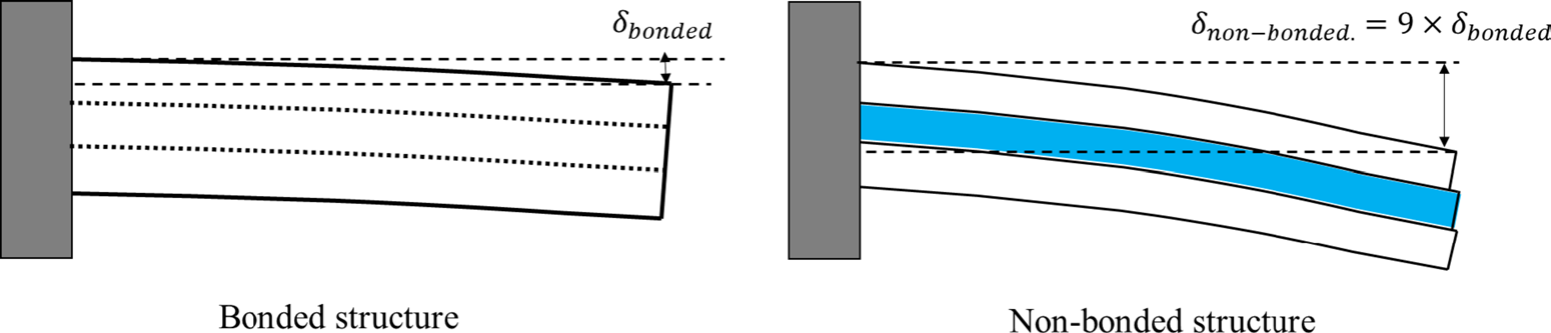
Schematic illustration of deformation patterns in bonded and non-bonded restorations.

Overall, the results indicate that contact nonlinearity had a larger influence than material nonlinearity under the present loading conditions. This was because the stresses acting on amalgam and gold restorations did not exceed the yield strength of each material. Therefore, to observe the effects of high plastic stress and strain beyond the yield strength of amalgam and gold, additional studies on the effects of local stress concentration and repetitive loading are required. The reason underlying the non-linear contact conditions induced significant stress can be inferred from Fig. 8. Even if a vertical force is applied to the occlusal surface, flexion deformation occurs in the cusp of the tooth, as shown in Fig. 8. In composite and ceramic, to which bonded contact condition was applied, both the tooth and restoration were deformed together, as shown in Fig. 8 (a) and 8 (b), respectively. However, in the case of amalgam and gold, which allowed non-linear contact condition, the tooth and restoration were deformed separately, as shown in Fig. 8c and d, resulting in relatively high stress on the tooth structure. As illustrated in Fig. 9, bending stiffness is proportional to the cube of thickness (∝ t³); therefore, loss of structural continuity in non-bonded restorations can lead to approximately ninefold greater deformation compared with bonded three-layer systems of equivalent thickness. Based on this study, consideration of the non-linear contact condition of the restoration is important for evaluating the stress and mechanical behavior of the tooth–restoration complex. However, in this study, unlike in actual clinical practice, there is a limitation in that differences in the thickness or physical properties of bonding adherents according to restorative materials and differences in cavity design were not considered. Because the deformation or stress concentration of teeth may vary owing to these differences, further studies are needed to address these limitations.

In addition, the finite element model was limited to the crown portion and did not include the periodontal ligament, root structures, or alveolar bone, which may influence absolute stress magnitudes. Static loading conditions were applied, whereas cyclic or fatigue loading may better reflect clinical function. Furthermore, the cavity geometry was simplified, and adhesive or cement layers were not explicitly modeled. These simplifications were adopted to allow controlled comparison among restorative materials; therefore, the results should be interpreted as relative mechanical behavior within the constraints of the numerical model.

## Conclusion

In this study, the mechanical behavior of the tooth-class II restoration complex was investigated with various restorative materials (composite resin, ceramic, amalgam, and gold) using three-dimensional non-linear FEA. Two non-linearities–non-linear mechanical properties and non-linear contact conditions–were considered in amalgam and gold restorations.

The maximum total deformation of the restoration was found to be largest for composite resin, followed by amalgam, gold, and ceramic restorations in descending order.

The highest stress occurred in amalgam restorations, whereas the lowest stress occurred in ceramic restorations. In amalgam restorations, the stress generated in the enamel and dentin was 83% and 127% higher, respectively, than that in ceramic restorations. These results provide mechanical insight into previously reported clinical observations of crack formation in teeth restored with different materials but should be interpreted within the limitations of numerical modeling.

Non-linear contact conditions seem to have played a more critical role because higher stress occurred in amalgam and gold, as the stress acting on these restorations did not exceed the yield strength of each material. Based on this study, consideration of non-linear contact conditions may be important for evaluating the stress and mechanical behavior of the tooth–restoration complex.

## Supplementary Information

Below is the link to the electronic supplementary material.

Supplementary Material 1

## Data Availability

- The datasets generated and/or analyzed during the current study are available from the corresponding author on reasonable request. - All data generated or analysed during this study are included in this published article [and its supplementary information files].
