# Supplementary material for: Mechanical behavior of tooth-class II restoration complex with various restorative materials using linear and non-linear finite element analysis
Source: Sci Rep. 2026 Feb 21;16:10150. doi: 10.1038/s41598-026-40204-3 (PMC13022157; doi:10.1038/s41598-026-40204-3)
Supplement: Supplementary file 1 — Supplementary Material 1 [file 41598_2026_40204_MOESM1_ESM.pdf]

Mesh information

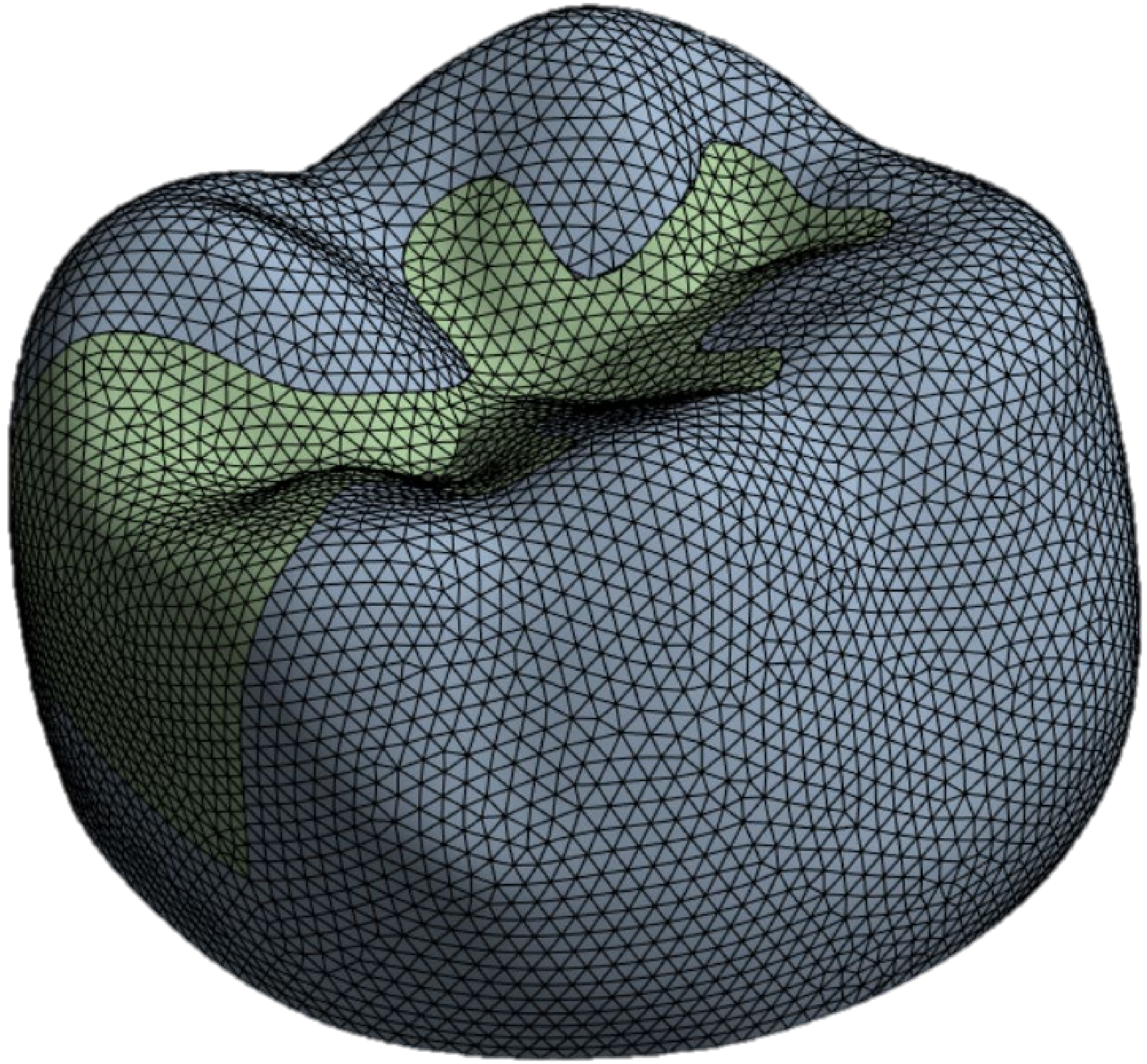

- Type: Tetrahedron
- Element order: Quadratic
- Avg. mesh size: 0.28 mm
- Elements: 73264
- Nodes: 125493

Boundary condition

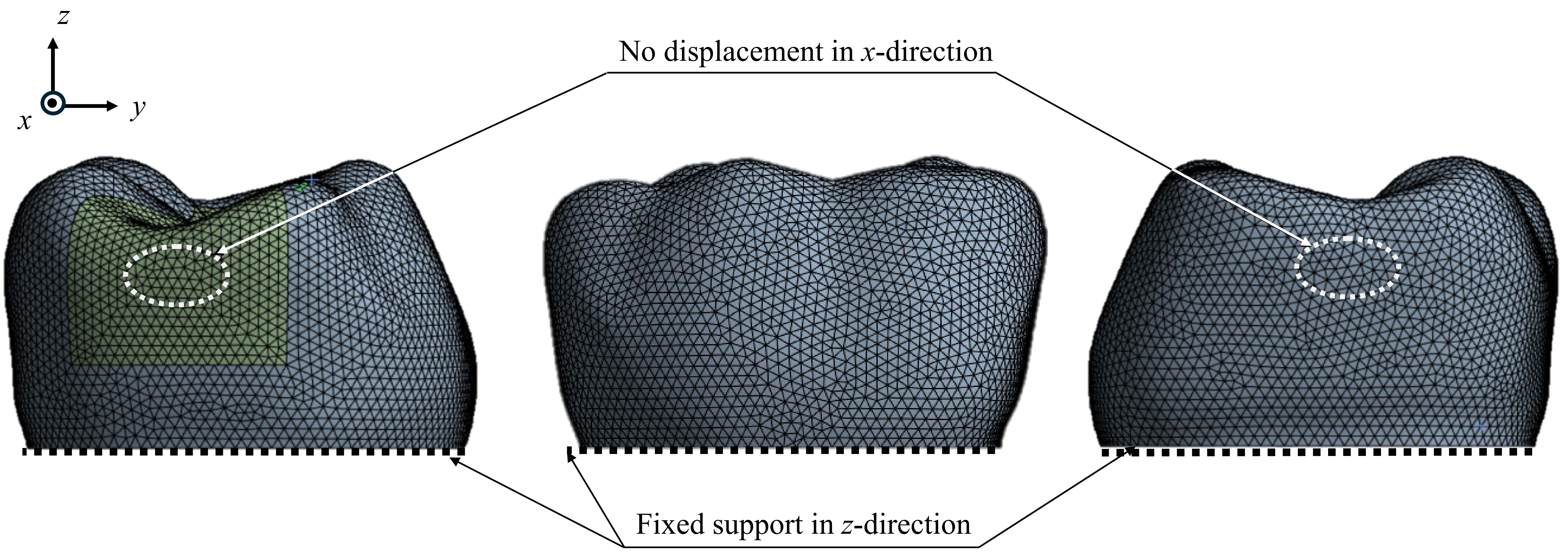

Loading condition

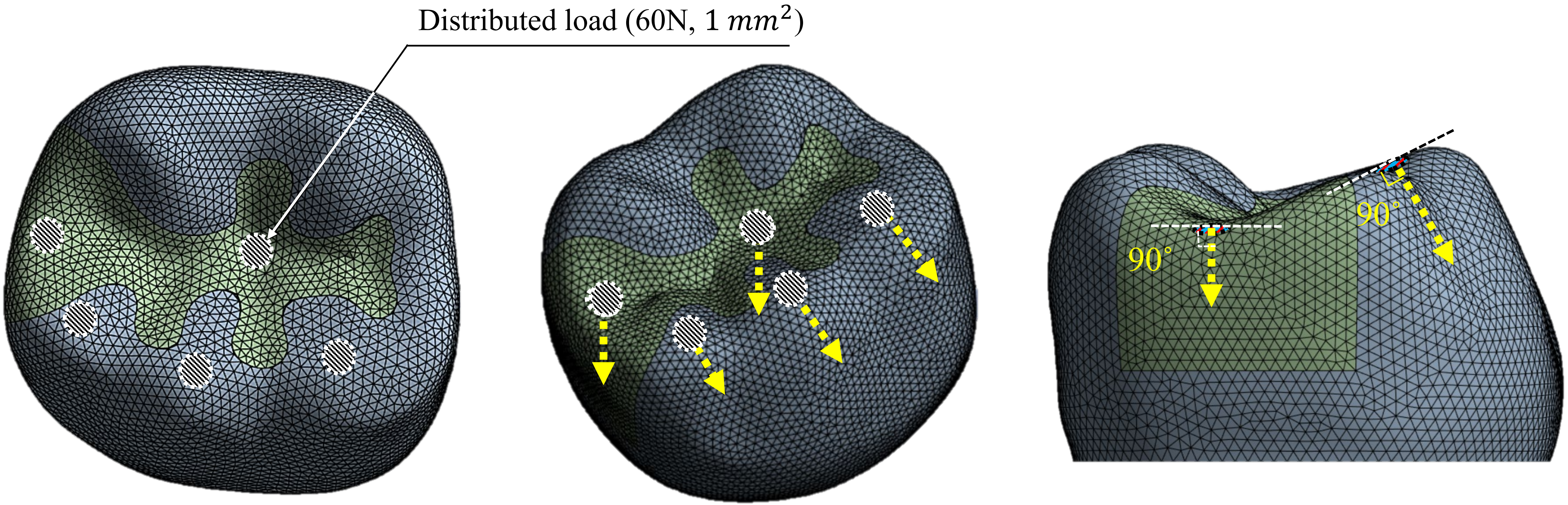

- Total occlusal force: 300N

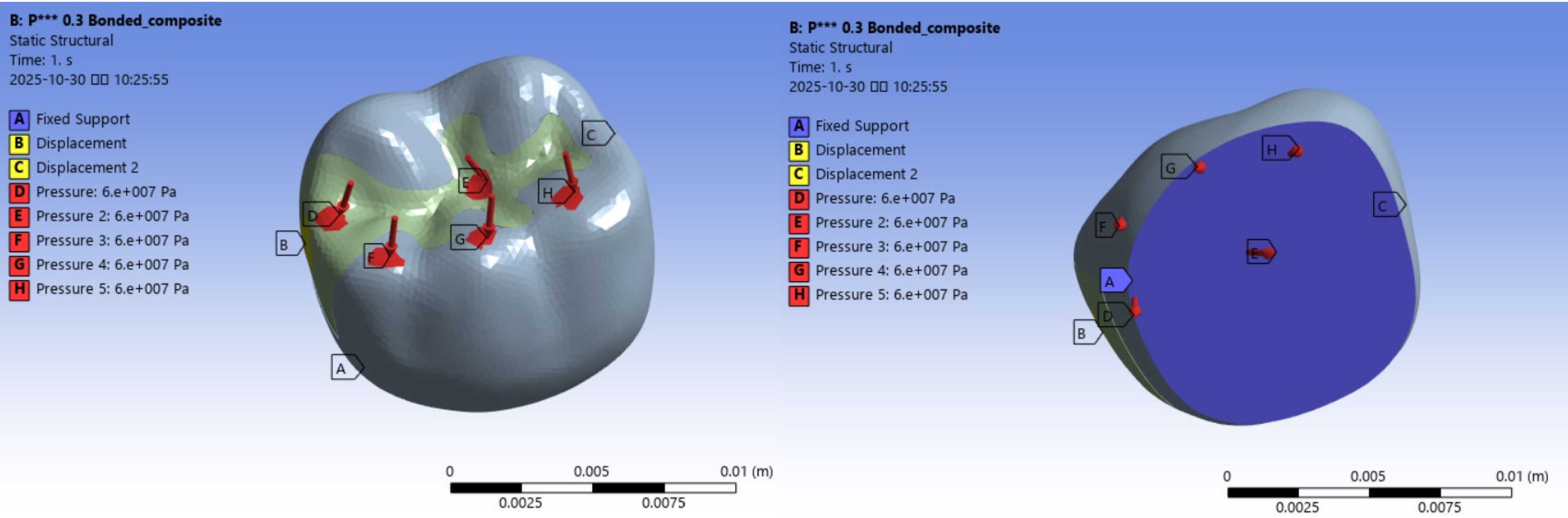
B: P*** 0.3 Bonded_composite
Static Structural
Time: 1. s
2025-10-30 □□ 10:25:55
A Fixed Support
B Displacement
C Displacement 2
D Pressure: 6.e+007 Pa
E Pressure 2: 6.e+007 Pa
F Pressure 3: 6.e+007 Pa
G Pressure 4: 6.e+007 Pa
H Pressure 5: 6.e+007 Pa
0
0.005
0.01 (m)
0.0025
0.0075
B: P*** 0.3 Bonded_composite
Static Structural
Time: 1. s
2025-10-30 □□ 10:25:55
A Fixed Support
B Displacement
C Displacement 2
D Pressure: 6.e+007 Pa
E Pressure 2: 6.e+007 Pa
F Pressure 3: 6.e+007 Pa
G Pressure 4: 6.e+007 Pa
H Pressure 5: 6.e+007 Pa
0
0.005
0.01 (m)
0.0025
0.0075
